# Editorial: Iodine in health and disease

**DOI:** 10.3389/fnut.2023.1260834

**Published:** 2023-08-15

**Authors:** Elisa Keating, Edgar Pinto, Agostinho Almeida

**Affiliations:** ^1^Unit of Biochemistry, Department of Biomedicine, Faculty of Medicine, University of Porto, Porto, Portugal; ^2^CINTESIS@RISE, Faculty of Medicine, University of Porto, Porto, Portugal; ^3^REQUIMTE/LAQV, Department of Chemical Sciences, Faculty of Pharmacy, University of Porto, Porto, Portugal; ^4^Department of Environmental Health, ESS, Polytechnic of Porto, Porto, Portugal

**Keywords:** iodine, iodine deficiency, iodine excess, childhood, pregnancy, thyroid hormones

Iodine is an essential micronutrient, used by the thyroid gland to synthesize thyroid hormones that perform pleiotropic functions in the human organism. These roles include stimulating somatic growth and regulating metabolism, cardiac function (1), and neurodevelopment, the latter through the control of synapse formation, neuronal migration, and myelination (2).

Iodine is supplied by food sources in several chemical forms: iodide (I^−^) and iodate (IO_3_^−^), generally in the form of sodium or potassium salts, molecular iodine (I_2_), and monoatomic iodine in association with proteins, the so-called organic iodine (3).

Natural food sources of iodine include seafood, dairy products, and eggs. However, these food sources may contribute differently to iodine intake, depending on the frequency of consumption but also on geological and climatic conditions and specific livestock practices in different regions of the world (4).

Additionally, artificial sources of iodine include iodized salt and food supplements, which provide potassium or sodium iodide or potassium iodate.

The extreme importance of iodine throughout the life cycle is evidenced by a set of diseases that result from iodine deficiency (the iodine deficiency disorders, IDD) and the consequent inadequate production of thyroid hormones.

The consequences of iodine deficiency largely depend on the stage of the life cycle and also on the severity of the iodine deprivation. For example, during pregnancy, severe iodine deficiency results in severe mental and growth impairments associated with deaf-mutism and spasticity (all features of cretinism); on the other hand, mild-to-moderate iodine deficiency *in utero* is associated with impairments in neurodevelopment revealed by a decreased intelligence quotient, impairment in fine motor skills, and also in verbal and non-verbal communication skills (5, 6).

The inadequate intake of iodine is currently a worldwide problem because, as already mentioned, the natural availability of this element depends on a series of geographical, geological, and climatic factors that escape direct human action.

The oceans are the largest reservoirs of iodide that, once transformed into elemental iodine, follows the water cycle and is supplied to the soil through rain. However, the iodine content of soils, and therefore of food crops, is greatly diminished due to rain leaching in areas of high rainfall. Soils deficient in iodine and, consequently, nutritional iodine deficiency are therefore more common in mountainous inland areas with high precipitation rates (6), but this deficiency is also detected in very rainy coastal zones, such as the Azores islands, Portugal.

All these conditions underlie the existence of endemic areas for iodine deficiency and the high global prevalence of IDD, regardless of the degree of local socioeconomic development.

In this context, the prevention of IDD through the promotion of adequate iodine intake was a global public health objective during the 1990s, motivating the WHO and UNICEF to recommend universal salt iodization. Recent data reveal that this strategy resulted in a decrease in the number of countries with iodine deficiency from 113 in 1993 to 20 in 2017 (7).

While iodine replenishment through salt iodization programs or iodine supplementation in populations with severe iodine deficiency has been shown to be effective in preventing IDD, this may not be the case in populations with mild-to-moderate iodine deficiency. On the other hand, these strategies have also precipitated the occurrence of circumstances of excessive iodine intake (8).

Despite all this, iodine deficiency in pregnant women remains prevalent. For example, in 2015, Zimmermann et al. reported that pregnant women in about 2/3 of European countries were iodine deficient (9).

The purpose of this Research Topic was to collect recent updates related to the role of iodine in health and disease. It includes four articles that address different aspects of this topic.

One emphasizes the occurrence of widespread iodine deficiency during pregnancy (2019 data), particularly in women not taking iodine supplements in Jiangsu Province, China. In this Chinese province, the average iodine content of iodized salt for household usage was lowered in 2012 due to concerns about excessive iodine intake. In this same paper, Wang et al. report that urinary iodine concentration (UIC) below 100 μg/L, corresponding to moderate-to-severe iodine deficiency, in the first trimester of pregnancy is associated with an increased risk of subclinical hypothyroidism later in pregnancy among euthyroid women.

On the other side of the iodine status spectrum, Candido et al. aimed to build a theoretical model that could estimate iodine intake from all food sources in childhood and pregnancy in a region of Brazil, a country with a history of excessive iodine intake resulting from excessive iodized salt consumption. This theoretical model, based on a healthy diet, estimated that daily intake of iodine by schoolchildren and pregnant women exceeds the recommendations. The authors propose that the developed model could be used to guide public policies for managing the population's iodine status.

Importantly, in addition to concerns about iodine deficiency and iodine excess updated by Wang et al. and Candido et al., respectively, Remer highlights, in an opinion article, the possibility that the increase in TSH levels as a result of improved iodine nutrition in populations with mild-to-moderate iodine deficiency is of a physiological rather than pathophysiological nature and that a general misinterpretation of these higher TSH levels has been documented, contributing to the probable over-prescription of thyroxine. This may motivate the search for new TSH reference values according to iodine status.

Lastly, Liu et al. propose that aging alters immune and inflammatory responses, as well as the mitochondrial function and cytochrome P450 metabolism of the thyroid gland, emphasizing that iodine replacement measures can differentially affect the population according to the life cycle stage.

Overall, the articles included in this Research Topic highlight that iodine deficiency or excess remain major public health issues that require close country- and life cycle stage-specific monitoring.

## Author contributions

EK: Writing—original draft. EP: Writing—review and editing. AA: Writing—review and editing.
